# Current State of Celery Allergy: Is Discovering Api g 7 a Milestone in Diagnosing Celeriac-Allergic Patients?

**DOI:** 10.3390/ijms26125840

**Published:** 2025-06-18

**Authors:** Bernadetta Kosztulska, Zbigniew Bartuzi, Natalia Ukleja-Sokołowska

**Affiliations:** 1 Department and Clinic of Allergology, Clinical Immunology and Internal Diseases, Jan Biziel University Hospital No. 2 in Bydgoszcz, Ujejskiego 75, 85-168 Bydgoszcz, Poland; bernadetta-kosztulska@wp.pl; 2 Department and Clinic of Allergology, Clinical Immunology and Internal Diseases, Ludwik Rydygier Collegium Medicum in Bydgoszcz, Nicolaus Copernicus University in Torun, 87-100 Torun, Poland; zbartuzi@cm.umk.pl

**Keywords:** allergy, celery allergy, celery–mugwort syndrome, Api g 7, defensins, component diagnostics

## Abstract

Celery allergy is a common food allergy, particularly among the European population. Currently, several diagnostic methods are available, including multiplex assays, which are useful for identifying celery-allergic patients. However, all of these methods have certain limitations. Api g 7 is a newly identified celeriac allergen belonging to the defensin protein family. Its clinical relevance lies in the high risk of severe systemic reactions among patients sensitized to this molecule. Patients sensitized to Api g 7 are often co-sensitized to Art v 1, the major mugwort (*Artemisia vulgaris*) allergen, due to structural similarity between these two molecules. This molecular homology plays a key role in the pathogenesis of celery–mugwort syndrome. Although Api g 7may be a major celery allergen, none of the currently available commercial diagnostic tests are capable of detecting sIgE against it. This highlights the need for the development of new, commercially available diagnostic tools in allergology.

## 1. Introduction

Celery (*Apium graveolens*) is one of the most common causes of food allergy, particularly in European countries [1,2,3,4]. Up to 0.45% of the adult population in Northern Europe suffers from allergy to celery root [1]. It is also a frequent cause of food sensitization and food allergies in children, especially in areas endemic to birch pollen [2]. Allergic reactions to celery can manifest in various forms, from itching, rashes, and urticaria, to oral allergy syndrome, angioedema, to even severe anaphylactic reactions [5,6,7].

Therefore, allergy to celery root has been a popular subject of interest for many researchers. For instance, the term “celery allergy” yields 236 search results in PubMed, the National Library of Medicine’s free bibliographic database, which includes scientific articles published between 1948 and 2024. Despite the enormous growth of molecular methods over the past decades, precise diagnosis of celery allergy still remains a challenge for both researchers and clinicians [3,4]. One of the crucial developments in the diagnosis of celery root allergy may be the recently discovered allergen of celery, Api g 7 [8]. As previous research has shown, Api g 7 can be classified as another “major celery allergen”, with more than 50% of celeriac-allergic patients producing sIgE antibodies against this molecule [9]. Some patients hypersensitized to Api g 7 show negative test results with celery extract [1,9]. Many of them will develop severe systemic reactions after consuming even small amounts of celery [9]. Therefore, confirming hypersensitivity to Api g 7 would aid in establishing the correct diagnosis in many cases.

In this article, we summarize current diagnostic methods and explore the potential ways to improve molecular tests for detecting specific antibodies present in patients allergic to *Apium graveolens*.

## 2. Characteristics of Celery Allergens—Chemical Group, Thermostability, Cross-Reactions

Since 2003, seven allergens from *Apium graveolens* (celery) have been identified and described in the WHO/IUIS Allergens Database [8,10]. A summary of the celery allergens identified to date is provided in Table 1.

### 2.1. Api g 1

Api g 1 is a member of the pathogenesis-related protein family PR-10 [8,13], and it is present in the sera of 59% of patients allergic to celery in Central Europe [14]. As noted by Bohle et al., the homologous protein of Api g 1 is Bet v 1,the major allergen of birch pollen [13]. Api g 1, which consists of 146 amino acids, shares 40–41% sequence identity and 61% sequence similarity with Bet v 1 [9,15,16]. This structural similarity is responsible for allergic cross-reactions to celery in birch-pollen sensitized individuals. In many studies, Api g 1 has been referred to as the “major celery allergen” (a major allergen is defined as one that sensitizes the majority of allergic individuals). The prevalence of specific IgE binding to Api g 1 varies across studies, ranging from 59% to as high as 80% [9,17,18]. To date, two isoforms of Api g 1 have been identified: Api g 1.0101 and Api g 1.0201. The latter is the most common isoform in celery root and exhibits lower IgE-binding activity [16].

Api g 1 exhibits IgE-binding capacity across a range of pH values [12]. However, this major celery allergen shows low thermal stability—Api g 1 is thermolabile and loses its structure after heating to 100 °C for 30 min [19]. The exact influence of temperature on Api g 1 is pH-dependent [19].

Pathogenesis-related proteins (PR-10), such as Api g 1, are involved in the pathogenesis of many food allergic reactions, including oral allergy syndrome (OAS) [20,21]. Typical symptoms of OAS include itching of the mouth and throat, as well as swelling of the lips, palate, and tongue, which begins immediately after contact between the oral mucosa and the allergenic food [22,23]. In rare cases, approximately 1–2% of patients with OAS experience serious systemic reactions, including anaphylaxis [22]. One of the most well-known PR-10 allergens is Bet v 1, which is present in birch pollen (*Betula verrucosa*) [21]. Various clinical studies have demonstrated that PR-10 proteins are involved in numerous cross-reactions between food and pollens, contributing to the pollen-food syndrome [20,21]. Many patients sensitized to Bet v 1 exhibit symptoms after exposure to fruits containing homologous PR-10 proteins, such as apple, hazelnuts, peach, golden and green kiwifruit, plum, and peanut [21].

### 2.2. Api g 2

Api g 2 is a type 1 non-specific lipid transfer protein (LTP), discovered and described in 2009 [8]. LTPs family is another diverse group of proteins implicated in allergies to many fruits and vegetables, particularly in pollen-related food allergies [14,24]. Like other LTPs, Api g 2 appears to be associated with oral allergy syndrome (OAS) and gastrointestinal allergy symptoms [14]. In contrast to PR-10 proteins, LTPs are resistant to pepsin digestion and high temperatures [20]. Api g 2 can remain intact in the gastrointestinal tract due to its high thermostability and resistance to proteolysis, even though its primary sequence contains 19 potential pepsin cleavage sites [25]. Specific antibodies against Api g 2 have been shown to play a crucial role in cross-reactions to celery, birch, and mugwort [25]. Api g 2 is predominantly expressedin celery stalks and is not present in the celery tuber [14,25].

Allergy to LTPs, such as Api g 2, was previously classified as a form of pollen-food syndrome. However, in light of recent data, this terminology may be imprecise. In patients with pollen–food syndrome, sensitization to pollen—such as *Artemisia vulgaris*,which contains the non-specific LTP (nsLTP) Art v 3—can lead to cross-reactivity with structurally similar allergens, such as Api g 2 from celery, which also belongs to the nsLTPs protein family [26]. However, what the authors consider crucial is that pollen proteins are not the only primary allergens involved in LTPs-related reactions. Pru p 3, the major peach allergen, can also trigger cross reactions with other foods containing proteins from the non-specific lipid transfer protein (nsLTPs) family [26,27,28]. Understanding the precise mechanism and interactions is highly important due to the clinical manifestations of LTP syndrome. Although it may present as oral allergy syndrome (OAS), many cases—particularly those involving LTPs and cofactors such as physical activity or non-steroidal anti-inflammatory drugs—can result in severe reactions, including anaphylaxis [29,30].

### 2.3. Api g 3

This chlorophyll a-b binding protein (chloroplast), expressed in *Apium graveolens*, is rarely described in the literature [8]. It is important to note that no commercial tests are currently available for the detection of Api g 3. This may explain its absence from most medical literature databases.

### 2.4. Api g 4

Approximately 23–41% of patients allergic to celeriac exhibit specific IgE antibodies to Api g 4 [14]. Like Api g 1, this celery profilin contributes to immunological cross-reactions, particularly the co-occurrence of birch pollen and celery allergies [13]. For example, Api g 4 shares 79.9% amino acid sequence identity with Bet v 2, a birch pollen allergen [15]. Profilins are common panallergens—anti-actin-associated proteins found in all eukaryotic cells—and are implicated in cross-reactivity with fruits, vegetables, nuts, legumes, and latex [31,32]. Bet v 2 appears to be the primary sensitizing allergen in profilin hypersensitivity [31]. It is estimated that 10–20% of pollen-allergic individuals produce antibodies against profilins [33]. Symptoms typically include OAS.

Clinical evidence suggests that patients sensitized to profilins are more likely to experience allergic rhinitis, severe rhinoconjunctivitis, and asthma compared to individuals without this sensitivity [31].

### 2.5. Api g 5

Api g 5, the only known celery allergen belonging to the high molecular weight (HMW) allergen group, is an FAD-containing oxidase [8,34]. It consists of a mixture of two polypeptides (with molecular weights of 53 and 57 kDa) that contain MUXF3-type N-glycans [34]. Up to 42% of patients allergic to celeriacs appear to be sensitized to Api g 5 [19]. Both Api g 5 and Api g 4 are thought to play important roles in cross-reactivity between celery and birch pollen [14]. Furthermore, Api g 5 is implicated in additional cross-reactions, primarily involving grass pollen and mugwort [34,35]. The similarity in N-terminal amino acid sequences between Api g 5 and a 60 kDa fennel allergenis believed to contribute to the mugwort–celery–spice syndrome [35].

In addition, the glycosylated form of Api g 5 has the capacity to bind IgE antibodies associated with celery allergy, whereas the deglycosylated isoform does not exhibit this property [4].

### 2.6. Api g 6

Another celery allergen, in addition to Api g 2, that belongs to the LTP protein family is Api g 6 [8]. Two subgroups of non-specific lipid transfer proteins (nsLTPs) are distinguished: nsLTPS-1 (with a molecular mass of approximately 9kDa) and nsLTPS-2 (16 kDa) [36]. Api g 6, a representative of the nsLTPS-2 subgroup, is found—like most celery allergens—in the celery tuber [8,36]. In contrast, Api g 2, an nsLTPS-1 protein, is present in the celery stalk [14,25].

In a 2013 study conducted by Vejvar et al., which included 32 patients with clinical reactions to *Apium graveolens*, approximately 37. 5% produced sIgE antibodies against Api g 6 [36]. Furthermore, the authors demonstrated that Api g 6 exhibits significant resistance to gastrointestinal digestion and heat, although it is not as thermostable as Api g 2 [36]. Like other LTPs, Api g 6 can trigger severe systemic reactions, including anaphylaxis [37]. Notably, this allergen is available in a commercial diagnostic test (ALEX).

### 2.7. Api g 7

This recently identified molecule may influence the approach to celery root sensitization. Api g 7, a defensin-like protein, was discovered and described by Wangorsch et al. in 2021 [1,8]. Its molecular structure plays a significant role in cross-reactivity among sensitized individuals [9,10]. This novel celery allergen appears to be a highly thermostable protein, with a melting temperature of approximately 100 °C [38]. Allergic reactions have also been reported following the consumption of cooked celery tuber in patients sensitized to celeriac defensin [9]. Given its frequency of hypersensitivity, Api g 7 may be classified as a major celery allergen [9,11]. In a study conducted by Balmer-Weber et al., about 52% of 79 patients produced sIgE antibodies against Api g 7 [9]. This newly identified celery allergen is likely to play a key role in advancing the diagnosis of food allergy. Its molecular similarity to other defensins may contribute to cross-reactivity and hypersensitivity to plants and foods containing defensin allergens, such as mugwort [1,9,10]. As previously mentioned, pollen-food syndrome can be triggered by various protein families, such as nsLTPs, profilins, PR-10, and defensins [39]. Therefore, the celeriac defensin Api g 7 is another molecule involvedin the pathogenesis of pollen-food allergy syndrome (PFAS). In this context, the condition is often referred to as celery–mugwort–spice syndrome or celery–birch–mugwort–spice syndrome (in patients with additional sensitization to the birch allergen Bet v 1 profilin) [9,40]. It should be noted that Api g 7 is not the primary allergen responsible for initiating allergic reactions in this group of patients. To date, no cases have been reported of individuals with PFAS who are allergic to celery without concomitant sensitization to pollen allergens [9].

Interestingly, the discovery of Api g 7 has demonstrated that not all celery-allergic patients test positive for celery extract [1,9]. It has been shown that Api g 7 may contribute to false-negative results in diagnostic tests based on whole celery extract [9]. In such cases, patients who are monoallergic to Api g 7 may be misclassified as not sensitized to *Apium graveolens*. This highlights the need for cautious interpretation of standard diagnostic tests, particularly given that sensitization to Api g 7 increases the risk of severe systemic reactions, such as anaphylaxis, by a factor of six compared to celery-allergic patients who are not sensitized to Api g 7 [9]. Unfortunately, despite its clinical relevance, Api g 7 is not detectable by any currently available commercial tests, including ISAC, ImmunoCAP, or ALEX.

## 3. Defensins and Their Role in Cross-Reactivity in Celeriac-Allergic Patients

In 1984, Wuthrich et al. observed that many patients sensitized to celery also exhibited allergic reactions to mugwort (*Artemisia vulgaris*) and consequently coined the term “celery-mugwort syndrome” [10]. In a 2022 study, Wangorsch et al. also reported that patients with celery hypersensitivity produce sIgE antibodies against Art v 1, a mugwort defensin [1]. The authors analyzed sera from eight patients allergic to celery—six of whom underwent a double-blind placebo-controlled food challenge (DBPCFC) with positive results, while the remaining two did not undergo DBPCFC due tohistory of anaphylactic reactions—and assessed their ability to IgE binding to recombinant celeriac defensin rApi g 7 [1]. All examined patients (100%) with a celery allergy showed sensitization to rApi g 7 [1]. Surprisingly, not all celery-allergic individuals test positive for celery extract [1,9,11]. For example, in a study conducted by Ballmer-Weber et al., among 12 patients allergic to celery and sensitized to Api g 7, only one tested positive for sIgE to celeriac extract using the ImmunoCAP (ThermoFisher Scientific, Uppsala, Sweden) [9]. Similarly, in the aforementioned study by Wangorsch et al., 62.5% of patients who produced sIgE antibodies against Api g 7 had negative ImmunoCAP test results for celeriac extract [1]. Even considering the limitations of these studies—such as small study groups and questionable statistical significance—they highlight a concerning bias in currently available commercial diagnostic methods. It is likely that Api g 7 is underrepresented in commercially produced celery extract [1,9]. Nevertheless, beyond this limitation, other clinically relevant aspects of Api g 7 include its potential for cross-reactivity with defensin-like proteins, which arises from its molecular structure.

Defensins are a diverse group of polypeptides synthesized by plants and animals, including humans [38,41]. In plant cells, in particular, they play a crucial role in cellular immunity. As molecules expressed in peripheral cell layers, they not only help combat bacterial and fungal infections, but also participate in regulating growth processes and cellular responses to stress [38]. Interestingly, a very similar phenomenon occurs in the human body. α-defensins produced by human neutrophils are involved in inflammation and immune responses, including the synthesis of IL-8. Moreover, they induce histamine secretion from mast cells and are likely associated with the pathogenesis of asthma [40]. Due to their widespread occurrence, defensin proteins are likely to cause allergic cross-reactions. According to the WHO/IUIS, 20 members of the large defensin family have been identified as allergens to date [38]. A notable example of a defensin-like protein is Art v 1, the major allergen of mugwort (*Artemisia vulgaris*) [42]. Approximately 95% of patients allergic to mugwort pollen exhibit sensitization to Art v 1 [40,43]. Art v 1 is localized in the cell wall [40]. It consists of galactose and arabinose and has a molecular mass estimated to be between 24 and 28 kDa [44,45]. This mugwort glycoprotein is formed by the fusion of two domains: a hydroxyproline-rich C-terminal domain and a cysteine-stabilized N-terminal defensin domain [43]. Naturally occurring Art v 1 (nArt v 1) undergoes extensive posttranslational modifications that result in significant structural changes [40]. The proline-rich C-terminal region is O-glycosylated either by the attachment of β-arabinofuranoside to hydroxyproline residues or by linking hydroxyproline to arabinogalactan, which consists of short β1,6-galactan chains [44,45]. The exact carbohydrate composition and complex posttranslational modifications may be factors that distinguish natural Art v 1 from its recombinant form (rArt v 1), which is typically produced in *Escherichia coli* [43]. IgE antibodies synthesized by a specific subgroup of patients have been shown to bind exclusively to natural Art v 1, but not to the recombinant form [44,45]. However, the C-terminal region is likely not involved in cross-reactivity with food allergens [38]. Plant defensins share a conserved cysteine-stabilized αβ-motif, typically consisting of eight conserved cysteine residues forming four disulfide bridges [38,40,45]. It was first described in 1991 by Kobayashi et al. [46]. This structural motif confers high resistance to temperature, pH changes, and the action of digestive proteases [45]. Defensins found in pollen (such as those from mugwort, ragweed, or feverfew) contain a polyproline-rich region, whereas food-derived defensins (from celery, peanuts, or soybeans) lack the C-terminal polyproline linker [38]. As previously mentioned, Art v 1 is considered a potential factor in certain allergic syndromes, such as mugwort–celery–spice syndrome or mugwort–chamomile syndrome, the latter of which primarily manifests with respiratory symptoms [9,41]. To date, several molecules from the defensin protein family have been identified as food allergens—Api g 7, a molecule derived from celeriac tuber, as well as Ara h 12 and Ara h 13, which are antifungal proteins primarily involved in immune responses against *Cladosporium* and *Alternaria*, and have been associated with severe allergic reactions to peanuts [9,47]. Gly m 2, a defensin-like protein found in soybeans, also exhibits a defensin-like structure; however, its exact role in the pathogenesis of soybean allergy remains unclear [38]. In the context of food allergy, particular attention should be given to Api g 7 and Art v 1. The structural similarity between the defensin Api g 7 from celery tuber and the N-terminal defensin domain of Art v 1 from mugwort pollen—these two allergens share approximately 60% sequence identity—leads to cross-reactivity and contributes to the clinical manifestation of mugwort–celery–spice syndrome [1,9,41,43]. In a study performed by Wangorsch et al. (2022) [1], a detailed graphic comparison of Api g 7 and Art v 1 protein structure similarity was presented in Figure S2/Appendix of the study [1]. It should be noted that other molecules also contribute to the pathogenesis of mugwort–celery syndrome, such as Api g 5 (in conjunction with the mugwort profilin Art v 4) and Api g 2, a non-specific lipid transfer protein (nsLTP), which cross-reacts with Art v 3, another nsLTP from mugwort pollen [9,11,14,26,29,30,35]. Respiratory allergy seems to be the factor that releases the cascade of allergic reactions. Ballmer-Weber et al. reported that there are no celeriac-allergic patients who are not also allergic to mugwort or birch, suggesting that sensitization to Art v 1 is likely primary in this group [9]. Furthermore, as noted by Wangorsch et al., sensitization to Art v 1 may fully inhibit IgE binding to Api g 7—indicating that Art v 1 outcompetes Api g 7 for IgE binding. This was demonstrated using the ELISA cross-inhibition method [1].

It is likely that many defensin-related proteins in food allergens have yet to be identified. For example, cases of severe allergic reactions to mango have been reported in association with a protein structurally similar to Art v 1, although no mango defensin has been characterized to date [38,48]. Considering this, additional clinical evidence of cross-reactivity between celeriac Api g 7 and other plant-derived food sources can be anticipated in the coming decades.

## 4. Diagnostic Methods of Allergy to Celery: Current Possibilities and Limitations

As previously mentioned, celery allergy is relatively common, particularly among the European population [1,11]. Therefore, accurate diagnosis—especially the identification of patients at high risk for severe systemic allergic reactions—is essential. To date, as with many other food allergies, only a limited number of diagnostic tools are available. The comparison of diagnostic tests useful in confirming celery allergy is presented in the Table 2.

Skin prick tests (SPTs) with food extracts—including celery extract—are among the first steps in basic allergy diagnostics. They are easy to perform and cost-effective [48,49]. When properly administered, SPTs yield reliable diagnostic results, as measured by both sensitivity and specificity [49]. The detection rate of SPTs for celery allergy is approximately 53% [49]. However, SPTs are not suitable for certain patient groups, such as individuals with atopic dermatitis, particularly those with extensive skin lesions [50]. Additionally, some patients with celery allergy—including those sensitized to the defensin Api g 7—may receive false-negative SPT results [9,11].

Modern allergy diagnosis should be based on the detection of specific IgE antibodies to food extracts or allergen molecules, despite certain limitations of these tests [12,45,51]. Methods that analyze sIgE binding to specific epitopes of allergens are more specific than those measuring sIgE binding to whole allergen molecules [52]. Multiplex tests allow the simultaneous detection of various specific IgE antibodies against multiple allergens, which can be particularly beneficial for patients with multiple allergies [50]. Several commercial methods for measuring sIgE are currently available on the market. Polycheck tests (Biocheck GmbH, Münster, Germany), an example of a multiple antigen simultaneous test (MAST) immunoblot assay, detect sIgE against laboratory-prepared celeriac extract across several panels (Polycheck Food 10-IV, Polycheck Food 20, Polycheck Food 30) [53]. These tests achieve approximately 88.9–100% correlation with skin prick test (SPT) results [54]. Polycheck Atopic 30-II appears useful for basic screening of patients suspected of celeriac allergy, due to its ability to simultaneously detect specific IgE against both celery extract and mugwort pollen extract [53]. Although some studies have demonstrated that Polycheck has sensitivity and specificity comparable to ImmunoCAP (Jeong et al., 2012; Jang et al., 2009), the latter remains the more precise and reference method for diagnosing food allergies [54,55,56]. The ImmunoCAP test (Thermo Fisher Scientific, Upssala, Sweden) can be performed as either a singleplex or a multiplex assay (ImmunoCAP ISAC) [12,55]. The singleplex ImmunoCAP demonstrates the highest detection rate among commercially available methods—approximately 66%—while the multiplex ISAC assay provides a detection rate of about 59% [47]. The ImmunoCAP singleplex method detects specific IgE against standardized celery extract as well as against the molecular allergen Api g 1 [9,57]. Multiplex tests, such as ISAC and ALEX, enable the simultaneous detection of specific IgE against multiple allergens [50]. The ISAC array consists of 112 recombinant and natural allergens derived from more than 50 allergen sources [55]. Unlike the ImmunoCAP singleplex assay, ISAC is a semiquantitative method and may be affected by interference from IgG antibodies [12,55]. The latest multiplex assay, ALEX, which is based on nanotechnology, enables the detection of both total IgE and specific IgE against allergen extracts and allergen molecules (comprising 23% natural allergens and 77% recombinantly produced allergens) [12,50]. The currently available ALEX2 allows for the detection of specific IgE against 295 allergens (117 extracts and 178 molecules) [12]. By removing IgE antibodies against cross-reactive carbohydrate determinants (CCDs) during preparation, ALEX minimizes the risk of false-positive results caused by cross-reactivity between antibodies [55]. In a comparison of multiplex assays, both ALEX and ISAC include Api g 1 in their allergen panels; additionally, ALEX also includes Api g 2 and Api g 6 [12].

In a 2022 study, Diem et al. compared ALEX and ISAC for the diagnosis of pollen-associated food allergy [50]. When detecting specific IgE against PR-10 proteins and profilins, ALEX demonstrated 90.02% specificity and 81.47% sensitivity relative to ISAC [50]. For the Api g 1 molecule specifically, ALEX showed a sensitivity of 77.8% (range: 40–97.2%) and a specificity of 97.7% (range: 88.0–99.9%) compared to ISAC [50]. Although the number of patients with IgE levels >0.3 was nearly equal between the two methods (ISAC: nine patients; ALEX: eight patients), differences were observed in the number of patients showing IgE reactivity to Api g 1—10 patients with ISAC and 15 with ALEX [50].

In addition to specific IgE detection, other methods can also be useful in the diagnosis of celery allergy. For example, skin prick-by-prick testing with native celery (typically both raw and cooked) may be performed [11]. This approach can be particularly beneficial for patients with inconclusive results from standard diagnostic tests, such as those sensitized to the defensin Art v 1 from mugwort.

The basophil activation test (BAT), a flow cytometry-based assay, appears to be even more sensitive than specific IgE detection and can, therefore, be used to reduce the potential risk associated with performing oral food challenges [52]. Therefore, the use of BAT in patients potentially allergic to the celeriac defensin Api g 7 may be particularly beneficial. As noted by Bartha et al., BAT demonstrates good predictive value for serious systemic reactions, with 76% sensitivity, 74% specificity, and 75% accuracy in identifying patients with severe allergic responses to baked eggs [52].

The oral food challenge (OFC) is a time-consuming and costly procedure that carries the risk of inducing severe allergic reactions [52]. As a result, some patients may refuse to undergo it [11,47]. There are specific exclusion criteria for OFC, including a history of severe reactions such as anaphylaxis, severe atopic dermatitis, or patient refusal [51]. Nonetheless, the double-blind placebo-controlled food challenge (DBPCFC) remains the gold standard for diagnosing food allergy [48,52,58]. The EAACI guidelines recommend the use of oral food challenge (OFC) in the majority of patients, suggesting that the double-blind placebo-controlled food challenge (DBPCFC) should be reserved for cases where an open OFC may yield inconclusive results or when required for scientific purposes [52]. Performing OFC in patients suspected of having a celery allergy should be based on strict eligibility criteria, taking into account the potential risks associated with the procedure [11].

**Table 2:** Comparison of diagnostic tests useful in confirming celery allergy [12,49,50,51,52,53,54,55,57,59,60].

Method	Characteristic	Limitations
SPTs	Relatively low cost Easy to perform Rapid results Enable detection of IgE-mediated reactions in skin	May yield false-negative results in a significant number of patients. Not suitable for certain patients populations, such as those with extensive skin lesions (e.g., severe atopic dermatitis) Subject to variability; results may depend on the experience and technique of the performing staff.
Polycheck	A diagnostic test for detecting sIgE against celery extract (available in panels, such as Polycheck Food 10-IV, Polycheck Food 20, and Polycheck Food 30) Relatively low cost	Lower detection rate compared to SPTs Not suitable for precise allergy diagnosis
ImmunoCAP	Allows quantification of sIgE against both celery extract and the major allergen Api g 1.001 Quantitative method No interference from IgG Highest detection rate among commercial methods (approximately 66%)	Does not allow detection of sensitization to other celeriac allergens, including Api g 7. Relatively expensive when a large number of tests is required
ISAC	Detects sIgE against Api g 1 Semiquantitative method Detection rate of approximately 59% Includes the native Art v 1 component Multiplex assay—useful in diagnosing polysensitized (multi-allergic) patients	Does not allow detection of Api g 7 Semiquantitative method Potential interference from IgG High costs
ALEX	Detects sIgE against Api g 1, Api g 2 and Api g 6 Sensitivity of 77.8% and specificity of 97.7% compared to ISAC (for Api g 1) Includes the recombinant Art v 1 component Specificity comparable to ISAC (97.7%) Multiplex assay—useful for diagnosing polysensitized (multi-allergic) patients	Does not allow detection of sensitization to Api g 7 Lower sensitivity (77.8%) compared to the ISAC method High cost

Currently available diagnostic methods for celery allergy, as with other food allergies, have certain limitations. It must be acknowledged that different celery cultivars may contain varying proportions of specific allergens, and in some specimens, certain allergenic molecules may even be absent [35]. This underscores the importance of collecting detailed clinical information—such as how the triggering meal was prepared, where the allergenic product was purchased, and other relevant circumstances—during patient evaluation.

Another clinically relevant issue is that none of the currently available commercial tests—neither ImmunoCAP, ISAC, nor ALEX—can detect specific IgE antibodies against Api g 7. As previously mentioned, patients sensitized to Api g 7 may produce false-negative results when tested with celeriac extract. This limitation can have serious consequences, particularly given that sensitization to Api g 7 increases the risk of severe systemic reactions, such as anaphylaxis, by sixfold compared to celery-allergic patients who are not sensitized to Api g 7 [9].

## 5. New Sight on Celery Allergy Diagnostics: What Do We Know So Far?

To date, no commercial test is available for detecting IgE antibodies against Api g 7. Therefore, it is crucial to establish specific recommendations for diagnosing patients in this subgroup:

- Skin prick testing with native allergens may help confirm celery allergy in a broader patient population, including individuals sensitized to allergenic components not represented in commercially available extracts, such as Api g 7.Nonetheless, the practical limitations and logistical complexity of this approach must be acknowledged. Additionally, a subset of Api g 7-sensitized patients may yield negative skin prick test results even when using native allergens (both raw and thermally processed celery tuber), suggesting limited sensitivity in certain clinical contexts [11].
- Identification of Art v 1 sensitization using Polycheck or ImmunoCAP may help isolate a subgroup of pollen-allergic patients with a significant predisposition to sensitization to defensin-like proteins.
- Oral food challenge (OFC), particularly the double-blind placebo-controlled food challenge (DBPCFC), remains the gold standard for diagnosing celery tuber allergy. However, due to the potential risk of severe allergic reactions during the procedure, its use should likely be limited to patients with a high pre-test probability of clinical allergy.

Skin prick testing (SPT) with native celery allergen and oral food challenge (OFC) may be valuable diagnostic tools in patients with confirmed sensitization to the mugwort defensin Art v 1. However, both approaches have limitations. SPT with native celery can yield false-negative results [11]. OFCs are associated with several contraindications, including the risk of severe systemic reactions—particularly in patients sensitized to Api g 7, even among those with no prior history of anaphylaxis to celery tuber [9].

## 6. Summary

Api g 7 is a recently identified major allergen in celeriac, belonging to the defensin protein family. Unfortunately, it is not detectable using currently available commercial diagnostic methods. Patients with a particularly high likelihood of sensitization to Api g 7 are those with pollen allergy to the mugwort defensin Art v 1, due to the high degree of structural similarity between these two proteins. Allergy to celeriac defensin remains a significant challenge in clinical practice, particularly given that patients producing sIgE against Api g 7 may test negative for sIgE against commercial celeriac extract. Current evidence indicates that sensitization to Api g 7 is associated with an increased risk of severe systemic reactions. Therefore, the development of commercial assays capable of detecting sIgE against Api g 7 would be of considerable clinical value.

Further research is needed to identify additional risk factors for anaphylaxis and to better understand cross-reactivity mechanisms in patients sensitized to defensin-like proteins.

## Figures and Tables

**Table 1:** Allergens from *Apium graveolens* (celery) [8,11,12].

Allergen and Its Biochemical Name	Protein Family	Molecular Weight	Feasibility of Marking Using Commercial Methods
Api g 1	PR-10 (pathogenesis-related proteins)	16 kDa	Yes (ImmunoCAP, ALEX, ISAC)
Api g 2	nsLTP type 1 (non specific lipid-transfer protein)	9 kDa	Yes (ALEX)
Api g 3	Chlorophyll a-b binding proteins	28 kDa	No
Api g 4	Profilin	14 kDa	No
Api g 5	FAD-containing oxidase	58 kDa	No
Api g 6	nsLTP type 2 (non specific lipid-transfer protein)	7 kDa	Yes (ALEX)
Api g 7	Defensin-like protein	12 kDa	No

## Data Availability

No new data were created or analyzed in this study. Data sharing is not applicable to this article.

## Abbreviations

The following abbreviations are used in this manuscript:

LTPs	Lipid-Transfer Proteins
SPTs	Skin Prick Tests
OFC	Oral Food Challenge
DBPCFC	Double-blind placebo controlled food challenge
